# Career plans of primary care midwives in the Netherlands and their intentions to leave the current job

**DOI:** 10.1186/s12960-015-0025-3

**Published:** 2015-05-10

**Authors:** J Catja Warmelink, Therese A Wiegers, T Paul de Cock, Evelien R Spelten, Eileen K Hutton

**Affiliations:** Department of Midwifery Science, AVAG and the EMGO Institute for Health and Care Research, VU University Medical Centre, Amsterdam, the Netherlands; Midwifery Academy Amsterdam Groningen (AVAG), Dirk Huizingastraat 3-5, 9713 GL Groningen, the Netherlands; Netherlands institute for health services research (NIVEL), Utrecht, the Netherlands; Faculty of Health Sciences, McMaster University, Hamilton, Canada; Department of Medicine, Nursing and Health Sciences, Monash University, Melbourne, Australia

**Keywords:** Career, Intention to leave, Job satisfaction, Midwives, Organization of care, Primary care

## Abstract

**Background:**

In labour market policy and planning, it is important to understand the motivations of people to continue in their current job or to seek other employment. Over the last decade, besides the increasingly medical approach to pregnancy and childbirth and decreasing home births, there were additional dramatic changes and pressures on primary care midwives and midwifery care. Therefore, it is important to re-evaluate the career plans of primary care midwives and their intentions to leave their current job.

**Methods:**

All 108 primary care midwives of 20 selected midwifery care practices in the Netherlands were invited to fill out a written questionnaire with questions regarding career plans and intentions to leave. Bivariate analyses were carried out to compare career plans and work-related and personal characteristics and attitudes towards work among the group of midwives who indicated that they intended to leave their current job (ITL group) and those who indicated they had no intention to leave (NITL group). Significant predictors of ITL were included in the multiple binary logistic regression with ‘intention to leave’ as the dependent variable.

**Results:**

In 2010, 32.7% of the 98 participating primary care midwives surveyed had considered an intention to leave their current type of job in the past year. Fewer ITL midwives wanted to be a self-employed practitioner with the full range of primary care tasks and work full-time. Significant predictors of the primary care midwives’ intention to leave included a lower overall score on the job satisfaction scale (OR = 0.18; 95% CI = 0.06–0.58; *p* = 0.004) and being between 30 and 45 years old (OR = 2.69; 95% CI = 1.04–7.0; *p* = 0.041).

**Conclusion:**

Our study shows that, despite significant changes in the reproductive, maternal and newborn health service delivery that impact on independent midwifery practice, the majority of primary care midwives intended to stay in primary care. The absence of job satisfaction, and being in the age group between 30 and 45 years old, is associated with primary care midwives’ intention to leave their current job. Ongoing monitoring will be important in the future.

## Background

### Primary care midwifery in the Netherlands

Maternity care is all care related to pregnancy and childbirth; midwifery care is that part of maternity care that is provided by midwives. In the Dutch maternity care system, midwifery care is community-based care for women with low-risk pregnancies. The primary care midwife in the Netherlands is an independent medical professional and provides care for a healthy woman with a healthy pregnancy (low-risk) throughout her pregnancy, birth and puerperium and is autonomous in her actions and decisions. In this echelon system, in case of complications, the primary care midwife will consult or refer/hand over to an obstetrician in a hospital (secondary care), or an academic referral centre (tertiary care), where clinical midwives provide the direct care for the woman. In 2012, 84.9% of all pregnant women in the Netherlands received care in early pregnancy from a primary care midwife, 52.4% started labour with a primary care midwife and 30.4% of all births (*n* = 173 099) occurred with a primary care midwife at home or in a hospital setting [1]. Almost all (95%) of the primary care midwives work in group practices, often with two or three colleagues, as self-employed practitioner, as employee in a partnership or as locum (a substitute midwife) [2]. Each practice provides 24/7 care through office hours and on-call hours of the midwives. Practice assistants are clerical staff and can help the midwife with administrative and organizational tasks. There are no workload ‘norms’ for primary care midwives, because most of them are self-employed, meaning they can decide for themselves how many clients they want to accept and how much time they want to spend with each client. That means there is no workable definition for full-time or part-time working in midwifery care in the Netherlands [3]. However, if 36–40 h a week is considered full-time, on average the independently self-employed midwife works 0.75 full-time equivalent (FTE) [2]. Most midwives in the Netherlands work independently in primary care, but in recent years, there has been a shift towards clinical midwifery [4]. In 2010, 72% of the 2612 practising midwives in the Netherlands worked in primary care in 519 midwifery practices [2]. A growing number (28% in 2011, compared to 16% in 2001) of midwives were employed as clinical midwives by hospitals and work under supervision of an obstetrician [2,5]. There is currently a debate on the reorganization of the maternity care system in the Netherlands. It may be a major turning point in the history of the midwifery care, going from an echelon system with primary, secondary and tertiary care to a more integrated maternity care system, where care from different providers is more integrated [6,7].

Students of the 4-year Dutch midwifery education at bachelor level are primarily trained to become independent primary care midwives. Most midwives in the Netherlands begin their careers working in locum positions for a few years before leaving this job type and usually becoming an employee or partner in an independent midwifery practice [8]. Various career changes are possible in primary care and can lead to a change in roles (such as when becoming an ultrasound operator, prenatal screener or preconception consultant) or a stronger focus on specific roles (such as providing prenatal and postpartum care but no intrapartum care). Career changes can also mean leaving primary care to get a job within the midwifery profession in different settings, like in the hospital as a clinical midwife and in midwifery education as a lecturer or a researcher, or leaving the midwifery profession altogether [2,8].

The increasingly medical approach to birth in the Netherlands is demonstrated by growing demand for medical pain relief, increasing use of inductions and augmentation in labour, and a related decreasing number of births in primary care [6,7]. It is likely that more pregnant and birthing women will receive care from medical specialists and clinical midwives in hospital in the future instead of from community-based primary care midwives. Currently, a growing proportion (28% in 2011, compared to 17% in 2002) of practising midwives are employed as clinical midwives by hospitals, where they work under the supervision of an obstetrician [2,5].

Over the last decade, besides the increasingly medical approach to pregnancy and childbirth and decreasing home births, there were additional dramatic changes and pressures on primary care midwives and midwifery care. The Dutch maternity care system was seriously criticized following the reports that the Netherlands had one of the highest rates of perinatal mortality in Europe. These figures were widely reported in various media, and often the automatic (unfounded) assumption was that the results were due to the unique Dutch organization of maternity care, leading to the suggestion that home birth and primary midwifery care were dangerous [7]. In that context, little is known about the midwives’ intention to maintain primary care practices. We do not know how many primary care midwives are considering leaving their current job in the light of these developments, nor factors associated with a desire to leave. Understanding the wishes of the current group of practising primary care midwives regarding their work situation can help further midwifery education and workforce planning. In this study, we wanted to examine the career plans of primary care midwives and their intentions to leave their current job.

### Career plans

Earlier studies [9-12] explored the career plans in general of primary care midwives in the Netherlands in 2001, 2002, 2003 and 2004 by asking about their preferred type of practice and preferred number of working hours in five years’ time. The results indicated that most primary care midwives wanted to stay in the midwifery profession and two out of three primary care midwives wanted to be (or remain) self-employed practitioners in private practice, while one in fourteen wanted to work as a clinical midwife. Two out of three midwives would like to work part-time (as most midwives in the Netherlands do [2]). A survey among graduating midwifery students in 2004 in the Netherlands [12] showed that most students preferred to start working full-time, but the majority of them planned to work part-time around the age of 30.

In our study, we wanted to explore the career plans of primary care midwives in the Netherlands in 2010 for the *near future* regarding the preferred type, nature and scope of their job in five years’ time and the way they would *ideally* organize their job with regard to ideal working hours and ideal practice type. We also wanted to make a comparison of these career plans between midwives who have an intention to leave their current job (ITL) in the last year and those who have no intention to leave (NITL).

### Intention to leave

Earlier studies of midwives’ intentions to leave their current job in the Netherlands indicated that on average 32% of midwives had been thinking about seeking other employment in the past year in the period from 2001 to 2004 [13]. No studies outside the Netherlands have reported on primary care midwives. Among related professions, Skinner et al. [14] showed in 2010 that one third (33.6%) of Australian nurses and midwives had at some time (no time frame stated) considered leaving the profession and in 2012 and 2013, Aiken et al. [15,16] showed that of the hospital nurses, 14% in the United States of America, 19% in the Netherlands and up to 49% in Greece intend to leave their job in the next year.

International research shows important predictors of intention to leave the health care profession include: a) *work-related* factors, such as work developmental opportunities, work environment and leadership [17-22]; b) *personal characteristics*, like being midcareer (between 30 and 45 years old) [23], being a single mother [24], having young children at home or a poor work–life balance [17,21,25,26]; and c) *attitudes towards work*, like experienced or perceived work pressure [17] and job satisfaction [17,18,25,27-30].

In our study, we wanted to assess the association between intentions of primary care midwives in the Netherlands to leave their job and factors associated with the likelihood of making this choice, such as a) work-related characteristics, including job type and region; b) personal characteristics, including age, family commitments and work–life balance; and c) attitudes towards work, like perceived work pressure and job satisfaction.

To summarize, three research questions were investigated in this study:

1. What are the career plans of primary care midwives in five years’ time regarding their near future and ideal work situation?
2. What are the differences in career plans between midwives who have an intention to leave their current job (ITL group) and midwives who have no intention to leave (NITL group)?
3. What is the relationship between the primary care midwives’ intention to leave their current job and their work-related and personal characteristics and attitudes towards their work?

## Methods

### Study design

This study is part of the DELIVER study [31], which is a multicentre prospective cohort study to evaluate primary care midwifery in the Netherlands with the main focus on quality, organization and accessibility of care.

### Recruitment and enrolment of study participants

Twenty of the 519 primary care midwifery practices in the Netherlands participated in the DELIVER study [31]. Practices were sampled using three stratification criteria: practice type (dual or group practice), level of urbanization (urban or rural area) and region (north, centre or south). All 108 midwives from the 20 participating practices were asked to complete a written questionnaire in May 2010. Midwives who did not complete and return the questionnaire received a reminder.

### Data collection

Regarding *career plans*, two multiple choice questions probed how participants viewed their work situation *in five years’ time*: “How do you see your work situation in five years’ time, with regard to the preferred type of job” (self-employed practitioner, employee, clinical midwife, locum, lecturer, researcher or not employed anymore) and “How do you see your work situation *in five years’ time*, with regard to the preferred nature and scope of your job” (primary care midwife with the full range of midwifery care, clinical midwife with the full range of midwifery care, ‘only prenatal and postpartum care and no intrapartum care’ or ultrasound operator in addition to or instead of midwifery care). On both questions, more than one answer could be given. Four questions (“What does your ideal midwifery practice look like, in terms of number of working hours and the ideal practice type (number of midwives, number of births and number of working hours of practice assistance”) probed how participants saw their ideal work situation regarding organization of their job.

To measure the *intention to leave*, we asked the question “Have you been thinking about seeking other employment *in the past year*” (yes/no) [32]. The questionnaire included *work-related* characteristics such as working years, job type (self-employed practitioners, employees or locum), practice type (group or duo practice), other work-related aspects (working in a single practice or in a number of practices, working also as an ultrasound operator, or in additional jobs other than practising midwife), level of urbanization (urban or rural area, or both), region (north, centre, south), country of midwifery education (in the Netherlands or abroad) and advanced postgraduate education (i.e. Masters degree) or additional training (ultrasonography, lactation consultancy). The questionnaire further included *personal characteristics* such as age (in years). Family commitments were measured with the questions “Do you have a partner” (yes/no) and “Do you have children at home” (yes/no) and with the follow-on question “Who is taking care of the children” (most of the time: me, my partner, both to the same amount, other). The question “Please indicate to what extent the combination of work and private life causes problems?” measured perceived problems in work–life balance using a 3-point rating scale (no problems, some problems, a lot of problems). Regarding *attitudes towards work*, perceived work pressure was measured based on the (five items) scale of Ruijters and Stevens [33] and overall job satisfaction (seven items) on the scale of Boumans et al. [34]. Both scales consist of general statements about the work situation in which the participants can indicate to what extent they agree on a 5-point Likert scale (strongly agree to strongly disagree). Another question, “To what extent are you satisfied with your work”, measured ‘satisfied with the work’ on a 4-point Likert scale. The question “Did you actually try to get another job?” (yes/no) measured job seeking activity.

### Data analysis

The variable ‘age’ was recorded into binary categories: the ‘mid-career group’ of between 30 and 45 years old vs. other ages, as suggested by Gurková et al. [23]. “Who is taking care of the children” was dichotomized in ‘me being the main caretaker’, versus other caretakers. ‘Perceived problems in work–life balance’ was dichotomized in ‘no problems versus some/many problems’. A new variable (mothers with work–life balance problems) was made for participants who stated yes to both questions: “Do you have children at home” and “Do you have some/many problems in work–life balance”. Scores scales were reversed so that higher scores represented higher perceived work pressure and higher job satisfaction. The variable ‘satisfied with your work’ was dichotomized in satisfied (score 1 ‘very satisfied’ and 2 ‘satisfied’) and less satisfied (score 3 ‘varying satisfied’ and 4 ‘not satisfied’).

All data were checked for outliers and extreme outliers were removed. Descriptive analyses were used to describe characteristics of the study population, and a comparison was made between the midwives who had an intention to leave their current job (ITL group) and midwives who did not had an intention to leave (NITL group). Bivariate analyses were carried out for comparing both groups with respect to work-related and personal characteristics, attitudes towards work and variables on career plans. For comparing means with respect to the continuous, normally distributed variables, we used Student’s *t*-test. If equal variances could not be assumed (Levene’s test), non-parametric equivalents were used. Pearson’s chi-squared tests (or Fisher exact tests for small sample size) were used for comparing the distribution of percentages of both groups with respect to categorical variables.

Of the scales ‘perceived work pressure’ and ‘overall job satisfaction’, the mean scale score for the whole group and two subgroups and Cronbach’s alpha were calculated.

Given our sample size of 98 cases, two predictors (independent variables) can be included in the multiple, hierarchical binary logistic regression model with the ‘intention to leave’ as the dependent variable model (the ratio of 30 cases for every variable [35]). Of all significant predictors of ITL (using a significance level of 10%), two variables that were independent from each other could be included. Of the scales, we preferred a reliability of the scale of Cronbach’s alpha > .70. We used Pearson’s *r* coefficient as a measure of statistical dependence between the predicting variables. The strength of the relationship between the variables was considered as moderate for *r* = .30 to .49 and strong for *r* = .50 to 1.0. For inclusion in the model, in case of multicollinearity, we preferred a multi-item-scale above a single item. The variables in the final model were presented as odds ratios (OR) with 95% confidence intervals (CI). In the final model, a two-tailed *p* value of .05 or lower was considered statistically significant. SPSS, version 21, was used for the analyses.

### Ethical approval and privacy issues

This study was carried out as part of the national DELIVER study, which obtained ethical approval by the Medical Ethics Committee of the VU University Medical Centre in Amsterdam, the Netherlands (WC 008–100). Participation of the midwifery practices in this study was voluntary, and consent to cooperate in this study was given. Privacy was guaranteed in accordance with Dutch legislation. Midwives’ anonymity was maintained by using anonymous midwife and practice identifiers.

## Results

### Baseline characteristics of participants

The questionnaire was returned by 99 of the 108 midwives (91.7% response rate).

One midwife did not answer some questions for our study and was thus excluded, leaving 98 midwives contributing to the study. Most participating midwives (95.9%) were working in group practices, 4.1% in duo practices and 0% in solo practice. Table 1 shows the sample characteristics of the group of 98 participating primary care midwives and of the 32 midwives (32.7%) in the ITL group and 66 midwives (67.3%) in the NITL group.

**Table 1:** **Sample characteristics of the whole group (** ***n*** **= 98) and of the 32 primary care midwives who intended to leave their current job (ITL) compared to the contrast group (NITL)**

	**Total (98 (100%))**	**ITL (32 (32.7%))**	**NITL (66 (67.3%))**	***p***
	***n*** **(%); mean ± SD**	***n*** **(%); mean ± SD**	***n*** **(%); mean ± SD**	
Work-related				
Job type				0.272
Self-employed practitioner	71 (72.4%)	26 (81.3%)	45 (68.2%)	
Employee	18 (18.4%)	5 (15.6%)	13 (9.7%)	
Locum	9 (9.2%)	1 (3.1%)	8 (12.1%)	
Group practice	94 (95.9%)	32 (100%)	62 (93.9%)	0.155
Other job types				
Working in a single practice	71 (72.4%)	21 (65.6%)	50 (75.8%)	0.292
Also practicing in another practice	4 (4.1%)	1 (3.1%)	3 (4.5%)	0.739
Ultrasound operator in another practice	8 (8.2%)	4 (12.5%)	4 (6.1%)	0.275
Also another job not as a midwife	16 (16.3%)	6 (18.8%)	10 (15.2%)	0.651
Level of urbanization				0.765
Urban	30 (30.6%)	11 (34.4%)	19 (28.8%)	
Rural	20 (20.4%)	7 (21.9%)	13 (19.7%)	
Urban and rural	48 (49.0%)	14 (43.8%)	34 (51.5%)	
Region				0.725
North	26 (26.5%)	7 (21.9%)	19 (29%)	
Centre	53 (54.1%)	19 (59.4%)	34 (51.5%)	
South	19 (19.4%)	6 (18.8%)	13 (19.7%)	
Education				
Midwifery education in NL	81 (84.4%)	27 (84.4%)	54 (84.4%)	1.000
Advanced/additional education	38 (38.8%)	10 (31.3%)	28 (42.4%)	0.287
Mean working years (±SD)	12.8 ± 9.9	14.5 ± 8.4	12.0 ± 10.5	0.203
Personal				
Mean age (±SD)	37.6 ± 10.8	49.4 ± 9.6	36.7 ± 11.4	0.238
Age between 30 and 45 years old	35 (35.7%)	18 (56.3%)	17 (25.8%)	0.003
Female	96 (99.0%)	30 (96.8%)	66 (100%)	0.320
Family commitments				
Having a partner	79 (81.4%)	26 (83.9%)	53 (80.3%)	0.673
Having children at home	54 (55.7%)	20 (64.5%)	34 (51.5%)	0.229
Be the main care taker	31 (31.6%)	10 (31.3%)	21 (31.8%)	0.955
Problems combining work and private life	73 (74.5%)	27 (84.4%)	46 (69.7%)	0.118
Mother with work–life balance problems (composite score)	47 (48.5%)	18 (58.1%)	29 (43.9%)	0.194
Attitudes towards work				
Perceived work pressure^a^ (mean scale score ± SD)	2.58 ± 0.664	2.78 ± 0.673	2.49 ± 0.644	0.044
Overall job satisfaction^b^ (mean scale score ± SD)	4.50 ± 0.418	4.28 ± 0.482	4.61 ± 0.339	0.001
Satisfied or very satisfied with work	81 (82.7%)	20 (62.5%)	61 (92.4%)	0.000

^a^The higher the score (range 1–5), the higher the perceived work pressure.

^b^The higher the score (range 1–5), the higher the job satisfaction.

### Career plans

Table 2 shows how the participating primary care midwives see their *future work situation* in five years’ time regarding their preferred *type* of job. Of all participants, 64.3% (*n* = 66) gave one preferred type of job; the others gave more than one preference. Three quarters (*n* = 75; 76.5%) of primary care midwives wanted to be (or remain) self-employed practitioners in their own midwifery practice, whether or not in combination with other job types. Significantly fewer midwives among the ITL group preferred to be self-employed in the past year compared to those with the NITL group (ITL = 62.5%; NITL = 83.6%; *p* = 0.022).

**Table 2:** **Career plans of the whole group (** ***n*** **= 98) and of the 32 primary care midwives who intended to leave their job (ITL) compared to the contrast group (NITL)** ^**a**^

	**Total (98 (100%))**	**ITL (32 (32.7%))**	**NITL (66 (67.3%))**	***p***
	***n*** **(%); mean ± SD**	***n*** **(%); mean ± SD**	***n*** **(%); mean ± SD**	
Preferred type of job in five years’ time^b^				
Self-employed practitioner	75 (76.5%)	20 (62.5%)	55 (83.6%)	0.022
Employee	15 (15.3%)	3 (9.4%)	12 (18.2%)	0.256
Lecturer	14 (14.3%)	7 (21.9%)	7 (10.6%)	0.135
Researcher	9 (9.2%)	5 (15.6%)	4 (6.1%)	0.124
Clinical midwife	7 (7.1%)	4 (12.5%)	3 (4.5%)	0.211
Locum	6 (6.1%)	3 (9.4%)	3 (4.5%)	0.389
Not working anymore	4 (4.1%)	3 (9.4%)	1 (1.5%)	0.101
Preferred nature and scope of job in five years’ time^c^				
Full range of primary care	80 (82.5%)	21 (67.7%)	59 (89.4%)	0.009
Ultrasound operator	27 (27.8%)	10 (32.3%)	17 (25.8%)	0.505
Full range of clinical midwifery	7 (7.2%)	4 (12.9%)	3 (4.5%)	0.205
Not employed anymore	4 (4.1%)	3 (9.7%)	1 (1.5%)	0.095
Only prenatal and postnatal care and no natal care	2 (2.1%)	2 (6.5%)	0 (0.0%)	0.101
Future and ideal work situation in five years’ time				
Full-time job	30 (30.9%)	5 (16.1%)	25 (37.9%)	0.027
Average part-time working time (hours per week)	26.9 ± 4.2	25.6 ± 4.2	27.1 ± 4.3	0.639
Ideal number of working hours (per week)	34.8 ± 6.3	32.3 ± 7.2	36.0 ± 5.4	0.015
Ideal number of midwives per practice	4.3 ± 1.5	4.2 ± 1.1	4.3 ± 1.6	0.862
Ideal amount of births per year	345 ± 153	323 ± 137	355 ± 160	0.338
Ideal average of practice assistance (hours per week)	27.1 ± 11.3	27.2 ± 9.6	27.0 ± 12.1	0.927

^a^“Have you been thinking about seeking other employment *in the past* year” (yes/no).

^b^“How do you see your work situation *in five years’ time*, with regard to the preferred type of job” (more than one answer possible).

^c^“How do you see your work situation *in five years’ time*, with regard to the preferred nature and scope of your job” (more than one answer possible).

Of the 32 midwives who have an intention to leave their current job (ITL group), 26 were self-employed midwives, 5 were employees and 1 was a locum. With regard to the preferred type of job, we looked in which direction they are moving. Eighteen of the 26 self-employed midwives wanted to remain a self-employed primary care midwife. Other mentioned preferred job types were ‘employee’ (2×), ‘locum’ (2×), ‘clinical midwife’ (1×), ‘lecturer’ (6×), ‘researcher’ (4×) and ‘not employed anymore’ (3×). Of the 5 employees in the ITL group, 2 wanted to become a self-employed primary care midwife and 1 preferred to remain an employee. Other preferred job types were ‘locum’ (1×), ‘clinical midwife’ (1×), ‘lecturer’ (1×) and ‘researcher’ (1×). The only locum in the ITL group saw ‘clinical midwife’ as the preferred type of job in five years’ time.

Table 2 also shows how the participants see their future work situation regarding the nature and scope of their job in five years’ time. Of the participants, 63.3% gave one preferred nature and scope of job; the others gave more than one answer. Eighty of the 98 midwives wanted to be a primary care midwife with the full range of midwifery care, with a significant difference between the two groups (ITL = 67.7%; NITL = 89.4%; *p* = 0.009). There were no significant differences between the two groups regarding the other preferred nature and scopes of job in the future.

Regarding their preferred *future* work situation, more NITL midwives are willing to work full-time (on average 36 to 40 h) (ITL = 16.1%; NITL = 37.9%; *p* = 0.027). There were no differences between the ITL and NITL groups on the average amount of working hours per week between the midwives who wanted to work part-time (range 16–32 h). There was however a significant difference on the *ideal numbers of working hours* per week between the two groups (ITL = 32.3 ± 7.2 h; NITL = 36.0 ± 5.4 h; *p* = 0.015). There were no significant differences between the two groups on their view of the ideal practice type (number of midwives, births and working hours of practice assistance). One midwife answered “90” to the question what the ideal number of midwives was. We excluded this from the calculation of the mean, because it was an extreme outlier.

### Intention to leave

The ITL and NITL groups did not differ on *work-related characteristics* or on *personal characteristics* (Table 1). Although there were no significant differences between the two groups in mean age, there was a difference (*p* = 0.003) when we looked at a specific age group: 56.3% of the midwives between 30 and 45 years old had an intention to leave their current job versus 25.8% of the midwives younger than 30 or older than 45. We also observed a moderate positive correlation between ITL and age group 30–45 years (*r* = 0.30, *p* = 0.003). The variable age group 30–45 years was included in the logistic regression model.

Table 1 shows significant differences between the ITL and NITL groups on the mean scale score on ‘perceived work pressure’, ‘overall job satisfaction’ and ‘satisfied with the work’. Correlation analyses demonstrated a positive correlation between ITL and ‘perceived work pressure’ (*r* = 0.20, *p* = 0.044) and a negative correlation with ‘overall job satisfaction’ (*r* = −0.37, *p* < 0.001) and ‘satisfied with the work’ (*r* = −0.37, *p* < 0.001). Looking at the above-mentioned predicting variables regarding attitudes towards work ‘overall job satisfaction’ showed a positive relationship with ‘satisfied with the work’ (*r* = 0.56, *p* < 0.001). We calculated the reliability of the scales ‘perceived work pressure’ and ‘overall job satisfaction’: Cronbach’s alpha of 0.687 and 0.791, respectively. None of the items would increase the reliability if removed. Regarding *attitudes towards work*, we chose to put ‘overall job satisfaction’ in the logistic regression model.

Table 3 shows that among the 98 midwives, the overall score on the job satisfaction scale (OR = 0.18; 95% CI = 0.06–0.58; *p* = 0.004) and being in the age group between 30 and 45 years old (OR = 2.69; 95% CI = 1.04–7.0; *p* = 0.041) were significant predictors of the primary care midwives’ intention to leave their job. In other words, midwives who were more satisfied with their job were much less likely to want to leave their job, controlling for the age factor in the model. However, the primary care midwives between 30 and 45 years old were 2.7 times more likely to have intentions to leave than midwives of other ages, taking job satisfaction into account. So, regardless of the level of satisfaction—even if they are very satisfied with their current job—primary care midwives between 30 and 45 years old are more inclined to consider seeking other employment than midwives in other age groups.

**Table 3:** **Binary logistic regression model analysing the relationship between the overall job satisfaction (5-point Likert scale), being between 30 and 45 years old and likelihood that a primary care midwife has an intention to leave her current job**

**Variable**	**(** ***β*** **)**	**SE**	**Wald** ***Χ*** ^**2**^	***p***	**Odds ratio**	***s***	
						**Lower**	**Upper**
Overall job satisfaction	−1.712	.598	8.188	.004	.180	0.056	0.583
Between 30 and 45 years old	.991	.485	4.172	.041	2.694	1.041	6.975
Constant	2.776	1.050	6.991	.008	16.059		

Seven midwives actually tried to get another job (outside primary care, but within the midwifery profession) as a clinical midwife, researcher or lecturer.

## Discussion

### Summary of the results

In our study, we wanted to find out what the career plans of primary care midwives in five years’ time would be and compare the characteristics of midwives who had an intention to leave with midwives who had no intention to leave their current job. We also wanted to examine the relationship between a primary care midwife’s intentions to leave the current job and her work-related characteristics, personal characteristics and attitudes towards work. Knowing the wishes of the current group of practising primary care midwives about their work situation may help further midwifery education and workforce planning.

Three quarters of the primary care midwives wanted to remain or become self-employed practitioners in their own midwifery practice in five years’ time and practice a full range of midwifery care. Consistent with previous studies in the Netherlands [13], one third of the participating primary care midwives in this study reported an intention to leave their current job. A number of differences in career plans were found between primary care midwives who have an intention to leave compared to those midwives who do not have an intention to leave. Fewer ITL midwives wanted to be a self-employed practitioner with the full range of primary care tasks and work full-time. There were no significant differences between the ITL and NITL groups regarding other preferred types or nature and scope of the jobs or in the way they would organize their ideal practice type. Of the work-related and personal characteristics and attitudes towards the job that were investigated, job satisfaction and ‘age group 30–45 years old’ were found to be related to the consideration to leave the job.

### Comparison with other research

#### Career plans

In our study, most of the primary care midwives wanted to remain or become self-employed practitioners in private practices in the near future, less than 5% of the primary care midwives did not want to be employed anymore in the near future, while 7.1% of all primary care midwives in our study wanted to leave primary care and work in a hospital as a clinical midwife in the future. These results were similar to the results reported by Wiegers and Janssen [13]. Regarding the way the (predominantly female) midwives would ideally organize their job, two thirds of the participants, and especially ITL midwives, wanted to work part-time in the future, like many women in the Netherlands. Before the arrival of the first child, one in two of all women in the Netherlands work full-time; after the arrival of the first child, this drops to one in five [24]. The mean age of the midwives is low compared to that of other groups of caregivers with an equivalent duration of education [13]. This may suggest that many midwives’ desire for part-time work may be related to the (expected) demands that come with having a family with (small) children [12,23].

#### Intention to leave

An earlier study of Wiegers and Janssen [13] on primary care midwives’ intentions to leave their job in the Netherlands showed similar results: one third (on average 32.3%) of the midwives had been thinking about seeking other employment in the past year in 2001 to 2004. The intention-to-leave ratio of the primary care midwives in our study is 3/10. While similar ratios for other countries are not available, they can be compared to those of hospital nurses. In Europe, the intention-to-leave ratio varies between 5/10 (i.e. in Greece, Finland and Poland) and 2/10 (i.e. in Netherlands and Norway) [16]. In our study, we did not find work-related factors as predictors of intention to leave the care profession. Relevant predictors came from the other two categories (personal characteristics and attitudes towards work). First, similar to a study by Gurková et al. [23], we found that midwives between 30 and 45 years old had a stronger inclination to leave the job than midwives in other age groups. Second, like other studies among care professionals, we found that job satisfaction was a significant predictor of midwives’ intentions to leave.

Almost 83% of primary care midwives in our study indicated that they were satisfied with their work. The 17% relative dissatisfaction compares favourably to job dissatisfaction among hospital nurses in Greece (56%), Ireland (42%) and China (45%) and is comparable to rates of dissatisfaction among hospital nurses in Sweden (22%), Norway (21%), Switzerland (21%) and the Netherlands (11%) [15,16,30]. It may be that the way in which midwifery care is organized in the Netherlands in 2010 with midwife-led care by independent, autonomous primary care midwives results in satisfied primary care midwives. This hypothesis is supported by our earlier exploratory work [36] that found factors contributing to job satisfaction among primary care midwives included direct contact with clients, supportive cooperation and teamwork with immediate colleagues, the organization of and innovation within their practice group and the independence, autonomy, freedom, variety and opportunities that they experienced in their work.

#### Importance of these findings

In labour market policy and planning, it is important to understand the motivations of people to continue in their current job or to seek other employment. Given the ongoing shift towards clinical midwifery in the Netherlands [4], understanding the wishes of primary care midwives regarding their work situation in the near future can help further workforce planning in maternity care. Primary care midwives have been under pressure of negative media, criticism, changing systems, etc., and therefore, it was important to re-evaluate their intention to leave their job. Our study shows that, despite some of the uncertainties about the future of primary midwifery care, the majority of primary care midwives intended to stay in primary care and this appears to be associated with job satisfaction. Ongoing monitoring will be important in the future, and any changes to the midwifery care system should take into consideration the perspective and preferences of all relevant professionals, including midwives.

### Strengths and limitations

Generalizability to primary care midwifery has been enhanced by the participation of midwifery practices from various geographical locations in the country. The practices in our study were a good representation for the Netherlands in terms of region and degree of urbanization. The response rate was high (91.7%). Wiegers and Janssen [13] also studied the question what career plans primary care midwives had regarding their work situation. The percentage (73.5%) of self-employed primary care midwives in the earlier study of Wiegers and Janssen in 2006 [13] was in accordance with the number of self-employed primary care midwives (72.7%) in this study.

There are limitations that must be considered in the interpretation of the results. The number of midwives is rather low (*N* = 98). By design, the midwives were selected from primary care practices only and are therefore not representative of the whole group of active midwives, including clinical midwives. Compared to Wiegers and Janssen [13], we see in our study a higher percentage of employed midwives (11.9% vs. 18.4%) and fewer locums (14.6% vs. 9.2%) and regarding the practice size, in our study, more midwives were working in group practices (46.9% vs. 95.9%), less midwives in duo practices (27.1% vs. 4.1%) and no midwife in solo practice (26.0% vs. 0%), so comparisons based on practice organization are tentative. There is also the issue of unknown unknowns: there is always the possibility of new variables that have not been considered or even defined, yet critical to the outcome. We did not take into account the ‘ease of movement’ as a factor commonly found in labour market models, like ‘job embeddedness’ (representing the midwives’ intra-organizational investments made both on and off the job) and the ‘job alternatives’ (defined as jobs available to midwives that they are qualified for and willing to accept) [37,38]. Nor did we focus on vocational interest as a predicting factor for leaving one’s job [39]. These factors will be important to include in future studies to develop deeper understanding. A disadvantage with our cross-sectional observational design is that it does not differentiate between cause and effect and does not provide an explanation for its findings. In this study, we identified associations that can be more rigorously studied in further research using other study designs.

It may be difficult to generalize the results to maternity care in other countries, because the Netherlands has a different setting of maternity care to most other Western countries. Furthermore, predicting leaving one’s job in rather individualistic cultures, such as the Netherlands, can be different from more collectivistic cultures [40]. The study of Ramesh and Gelfand [40] showed that person–job fit was a significant predictor of lower turnover in an individualistic country, whereas person–organization fit, organization links and community links were significant predictors of lower turnover in a collectivistic country. On the other hand, the results of our study can be relevant for countries that have a comparable maternity care system with autonomous midwives in private practice or countries that are implementing midwife-led maternity care [7,41].

### Recommendations

It would seem unrealistic to expect participants to never consider leaving their profession. Career changes are something ‘normal’ that a majority of midwives may consider at some point during their career. The high level of job satisfaction indicated that midwives generally enjoy their work in the primary care in the current system. When a different model of organization of midwifery care is envisioned in the Netherlands, for instance a system of more integrated maternity care (see the ‘Primary care midwifery in the Netherlands’ section), the high current job satisfaction among primary care midwives should be taken into account. A recommendation for potential further studies (quantitative as well as qualitative) would be to explore factors contributing to the job satisfaction of midwives. Furthermore, these studies should comprise all midwives including those who work in education, in research and in a hospital and those midwives who are not yet graduated or who are temporarily not working and might consider returning to primary care midwifery.

## Conclusion

Our study shows that, despite some of the uncertainties about the future of primary level midwifery care, the majority of primary care midwives intended to stay in primary care. Three quarters of the primary care midwives in the Netherlands wanted to remain or become self-employed practitioners in their own midwifery practice in five years’ time and practice a full range of midwifery care. Consistent with previous studies, one third of the participating primary care midwives in this study had an intention to leave their current job. This study found the absence of job satisfaction, and being in the age group between 30 and 45 years old, to be associated with primary care midwives’ intention to leave their current job. If and when a different model of organization of maternity care is envisioned in the Netherlands, the high current job satisfaction among primary care midwives should be taken into account.
